# Direct measurement of Ni incorporation into Fe_3_O_4_(001)†
†Electronic supplementary information (ESI) available. See DOI: 10.1039/c8cp02516a


**DOI:** 10.1039/c8cp02516a

**Published:** 2018-06-08

**Authors:** P. T. P. Ryan, Z. Jakub, J. Balajka, J. Hulva, M. Meier, J. T. Küchle, P. J. Blowey, P. Kumar Thakur, C. Franchini, D. J. Payne, D. P. Woodruff, L. A. Rochford, F. Allegretti, T.-L. Lee, G. S. Parkinson, D. A. Duncan

**Affiliations:** a Diamond Light Source , Harwell Science and Innovation Campus , Didcot , OX11 0DE , UK . Email: david.duncan@diamond.ac.uk; b Department of Materials , Imperial College London , South Kensington , London SW7 2AZ , UK; c Institute of Applied Physics , Technische Universität Wien , 1040 Vienna , Austria; d University of Vienna , Faculty of Physics and Center for Computational Materials Science , 1090 Vienna , Austria; e Physics Department E20 , Technical University of Munich , 85748 Garching , Germany; f Department of Physics , University of Warwick , Coventry , CV4 7AL , UK; g School of Chemistry , University of Birmingham , Birmingham , B15 2TT , UK

## Abstract

The normal incidence X-ray standing wave (NIXSW) technique has been used to follow the evolution of the adsorption geometry of Ni adatoms on the Fe_3_O_4_(001)-(√2 × √2)*R*45° surface as a function of temperature. Two primary surface region sites are identified: a bulk-continuation tetrahedral site and a sub-surface octahedral site, the latter site being preferred at higher annealing temperatures. The ease of incorporation is linked to the presence of subsurface cation vacancies in the (√2 × √2)*R*45° reconstruction and is consistent with the preference for octahedral coordination observed in the spinel compound NiFe_2_O_4_.

## Abstract 2

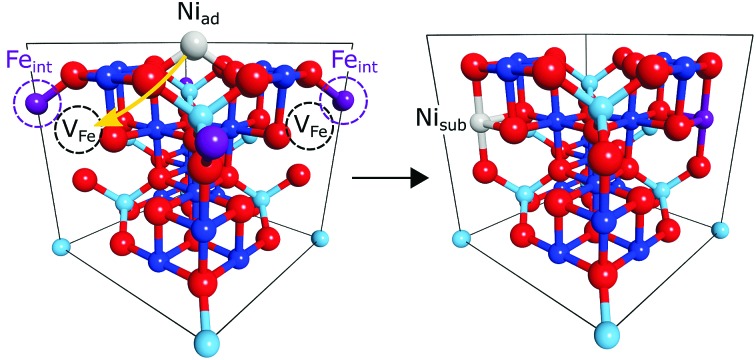
The normal incidence X-ray standing wave (NIXSW) technique has been used to follow the evolution of the adsorption geometry of Ni adatoms on the Fe_3_O_4_(001)-(√2 × √2)*R*45° surface as a function of temperature.

## Introduction

Maximising the surface area of a catalyst is one of the most common methods of enhancing its catalytic activity. As the size of the active component tends towards the nanoscale limit, a so-called “support material”, often a metal oxide, must be employed. The support prevents the sintering of the nanoparticles and can control their shape and size. However, the term “support material” becomes a misnomer for many reducible metal oxides, most famously in the case of supported Au nanoparticles,^1^–^3^ for which it is the interface between the nano-particles and the oxide surface that is the active site for CO oxidation. Such effects, termed “active supports” are widely reported in the literature,^4^ and are very common with iron oxide supports, often *via* Mars–van Krevelen (MvK) type reactions.^5^–^7^

In most active oxide supports, it is anion defects that dominate the surface chemistry. This has been most frequently reported for TiO_2_, on which, for example, oxygen vacancies enable water cracking to produce surface hydroxyl species;^8^ oxygen vacancies are also the active centres for photocatalytic reduction of NO to N_2_ and O_2_.^9^ In contrast, iron oxides are prototypical cation defect materials. For example, wüstite (FeO) almost never achieves stoichiometric FeO, with compositions of Fe_(1–*x*)_O being common in natural crystals,^10^ while the relative ease of transition between the two spinel iron oxides maghemite (γ-Fe_2_O_3_) and magnetite (Fe_3_O_4_) is a direct consequence of the facile creation and mobility of cation defects within their bulks. In this latter case, both iron oxides comprise fcc anion close packed structures that only differ in the arrangement of their cations; magnetite and maghemite contain the same number of tetrahedral cations (with respect to the O lattice), but maghemite contains only 5/6th of the octahedral cations. Thus, the origin of the active support behaviour of the iron oxides is ascribed to the ease of cation diffusion, both towards and away from their surfaces, in response to surface redox reactions.^11^

**Fig. 1:**
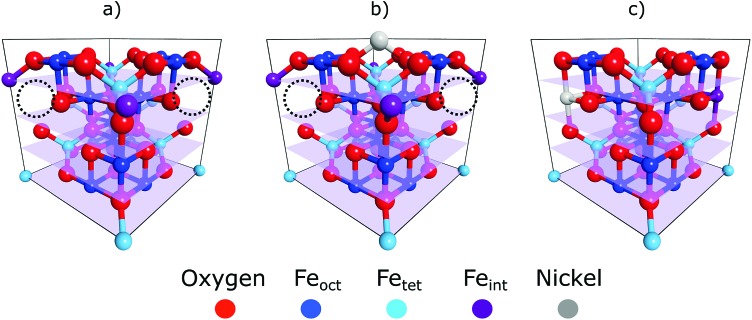
Unit cells for the (a) SCV reconstructed surface, (b) Ni adatom adsorption atop the SCV reconstruction and (c) Ni incorporation into the subsurface vacancies of the SCV reconstruction. Ni species are depicted in grey and the tetrahedral interstitial atom of the SCV reconstruction in magenta. The subsurface vacancies of the SCV reconstruction are indicated with the black dotted circles. Of note is the filling of both subsurface vacancies, in (c), by both the Ni atom and the interstitial to give back a bulk like unit cell or termination. The four transparent planes coincide with the four tetrahedral layers of the unit cells and the periodicity of the (004) reflection.

A prime example of how cation defect mobility dominates the surface chemistry of iron oxides, and the focus of this work, is the subsurface cation vacancy (SCV) structure of the Fe_3_O_4_(001) surface.^12^ The SCV structure was determined by quantitative low energy electron diffraction (LEED-IV),^12^ and explains the well-established (√2 × √2)*R*45° surface reconstruction of this substrate (Fig. 1a). This SCV structure includes two octahedral cation vacancies and one tetrahedral cation interstitial. Each cation vacancy has as its neighbour one of the two sub-surface oxygen atoms to which the interstitial cation is bound. Remarkably, this reconstruction allows a variety of metal adatoms to be stabilised into a dense array on the surface (up to ∼0.4 metal adatoms per surface unit cell)^13^–^15^ including highly catalytically-active precious metals, *e.g.* Pt and Pd.^16^,^17^ Such adatoms are suspected to be active sites in so-called “single atom catalysis” (SAC) – the *terminus ad quem* in the maximisation of catalyst surface area. The ability to have such a pure and uniform phase of monodispersed adatoms on a single crystal surface makes them accessible to a wide range of surface science techniques that cannot be applied to the polydispersed nanoclusters used in traditional catalysis. Thus, this model system could provide significant insight into support effects and catalysis in general.

In our prior work,^18^ the adatom adsorption site of Cu and Ag was determined, quantitatively, utilising the normal incidence X-ray standing wave (NIXSW) technique. Specifically, Cu and Ag adatoms were found to bridge two surface oxygen atoms in a site comparable to that of bulk tetrahedral iron, but at significantly different adsorption heights. The lateral adsorption site of these adatoms was originally proposed by inspection of STM images, in which they appear as strongly contrasting features along the [11[combining macron]0] direction between the Fe_oct_ rows of the surface. However, following deposition at room temperature, STM images of several first row transition metals adatoms, *e.g.* Ni and Co, exhibited additional dimmer features located slightly offset from the centred location of the adatom features.^15^ The number of these species increased dramatically with increasing coverage, or with post-deposition substrate annealing. In the case of Ni deposition, Ni 2p X-ray photoelectron spectroscopy (XPS) exhibited two peaks with a relative binding energy shift of ∼1.4 eV. Furthermore, an increasing concentration of these dimmer species led to the lifting of the reconstruction, as observed by LEED. DFT+U calculations^15^ predict that the lowest energy site for these Ni atoms is actually the octahedral vacancy site of the SCV reconstruction. This dimmer feature in the STM images has therefore been assigned to single atoms of Ni occupying the octahedral cation vacancy in the SCV structure.

However, STM measurements only provide an indirect measure of the surface topography and are strongly influenced by the electronic structure. More importantly, STM cannot image sub-surface topography and, as such, it is only the effect on the electronic structure at the surface of the potentially incorporated adatoms that is measured. Furthermore, our prior work^18^ has cast doubt on the accuracy of DFT+U calculations for modelling adatoms on the magnetite surface. As both the catalytic activity of metal nanoclusters on Fe_3_O_4_ supports, and the SCV structure that patterns the dispersion of these lone adatoms on the (001) surface of Fe_3_O_4_, are intrinsically linked to the cation vacancies, the step-wise incorporation of Ni atoms into these vacancies could provide a unique opportunity to probe the cation defect chemistry of magnetite. In this paper we confirm directly and quantitatively the presence of Ni atoms in the sub-surface, co-planar with octahedral iron, through our use of the chemical-state resolved NIXSW technique.

## Experimental details

All measurements were conducted in an ultra-high vacuum (UHV – ∼3 × 10^–10^ mbar) end-station on the I09 beamline at the Diamond Light Source. Beamline I09 has a double silicon(111) single crystal monochromator and a plane grating monochromator, providing access to both ‘hard’ and ‘soft’ X-ray energies, respectively. Specifically, we have used an incident photon energy of 1100 eV for all the soft X-ray photoelectron (SXP) spectra and of ∼2950–2960 eV for the NIXSW measurements.

Surface preparation was undertaken with conventional UHV sputtering and annealing procedures together with *in situ* surface characterisation of the prepared surfaces, before and after Ni deposition, by LEED and SXPS. These photoemission spectra, as well as those utilised for NIXSW analyses, were acquired using a VG Scienta EW4000 HAXPES hemispherical electron analyser (angular acceptance range ±30°), which was mounted perpendicular to the direction of photon incidence, in the plane of the incident radiation polarisation (linear horizontal).

The NIXSW technique exploits the standing wave that occurs due to the interference between the incident and reflected X-ray beams when a Bragg condition is satisfied in a crystal. This standing wave extends into and out of the bulk of the material and has a periodicity equal to the spacing between the Bragg planes, *d*_*hkl*_. The standing wave exists across a finite incident photon energy range within which its phase varies as a function of the incident photon energy, causing the nodes and antinodes of the standing wave to move relative to the Bragg planes. The photoemission intensity from an atom situated anywhere in or on the surface is therefore dependent on its location relative to the Bragg planes. Measurement of characteristic core level photoelectron yield profiles, as the incident photon energy is swept through the Bragg condition, therefore allows the height of the emitter atom above the corresponding Bragg planes to be directly determined. This modelling of the NIXSW data, based here on the dynamical diffraction theory of X-rays^20^–^22^ for the Fe_3_O_4_(004) reflection (a photon energy of 2955 eV and *d*_004_ = 2.10 Å), results in the determination of two fitting parameters, the coherent position (*P*_004_) and the coherent fraction (*f*_004_). In the case of single site occupation, the former parameter can be interpreted as the mean position of the emitter relative to the Bragg planes, expressed as a fraction of the spacing between these planes and is thus adimensional; the coherent fraction is indicative of the degree of order (or disorder) of the emitter's position relative to those planes, having a value of 1 for perfect order and 0 for complete disorder.

The (004) reflection was chosen, as the planes that contain tetrahedral and octahedral Fe-sites are separated by *d*_004_/2 and are thus antiphase to each other, corresponding to a difference in coherent position of 0.5. Here we have chosen to define the unit cell of the inverse spinel structure for Fe_3_O_4_ such that the coherent positions of the tetrahedral planes were 0 or 1, leading to a value for the octahedral planes of 0.5.

Photoelectron yield profiles were measured by monitoring the intensity variations and line-shape of the chemically resolved peaks observed in the Ni 2p_3/2_ core level. All peaks, except an observed satellite feature, were fitted with a convolution of a Gaussian and a Doniac–Sunjic line-shape. The satellite feature was fitted with a Gaussian lineshape alone. All NIXSW spectra were normalised to the drain current of the final hard X-ray mirror, which was assumed to vary linearly with the photon flux, whilst all soft XP spectra were normalised to the fitted areas of the Ni 2p_3/2_ SXP peaks (including the satellite feature). For the NIXSW profiles, a Ni 2p_3/2_ core level spectrum was acquired at regular steps over an 8 eV photon energy range, centred on the Bragg energy. These data were then fitted as described above, and in the ESI,† to deconvolute the contributions from each species observed in the spectrum. Thus, the data points in the NIXSW profiles are the integrated intensity of each separate peak in the Ni 2p_3/2_ core level spectrum measured as a function of the photon energy. With regards to spectra background subtraction, a detailed description of the XP spectra analysis is given in the ESI.†

Two polished Fe_3_O_4_(001) single crystals (±0.1°, from the Surface Preparation Laboratory and PI-KEM) were prepared *in situ* via several cycles of sputtering (Ar^+^, voltage: 1 keV, emission current: 3 mA, 10 min) and annealing (∼600 °C, 10 min). The annealing was alternated between annealing in UHV and in an oxygen partial pressure of 1 × 10^–6^ mbar; the latter step was included to prevent chemical reduction of the SCV reconstruction. The prepared samples showed a sharp (√2 × √2)*R*45° LEED pattern indicating the presence of SCV surface reconstruction.

Ni was deposited onto the prepared crystals using an Omicron EFM3 evaporator. The deposition rate was monitored by a water-cooled quartz crystal microbalance (QCM). Three depositions of Ni metal, with the sample held at room temperature, were conducted, with two of them being annealed to different temperatures (425, and 875 K). Specifically, the Ni coverages and annealing temperatures of the three preparations were: 0.2 ML, 300 K (room temperature); 0.2 ML, 425 K; and 0.5 ML, 875 K. Here, 1 ML is defined as one metal adatom per SCV reconstructed surface unit cell. A fourth deposition, with the sample held at ∼150 K, was also undertaken to a coverage of 0.3 ML. Ni 2p SXP spectra and a series of NIXSW measurements were taken for all four preparations.

## Results and discussion

### Soft X-ray photoelectron spectroscopy

**Fig. 2:**
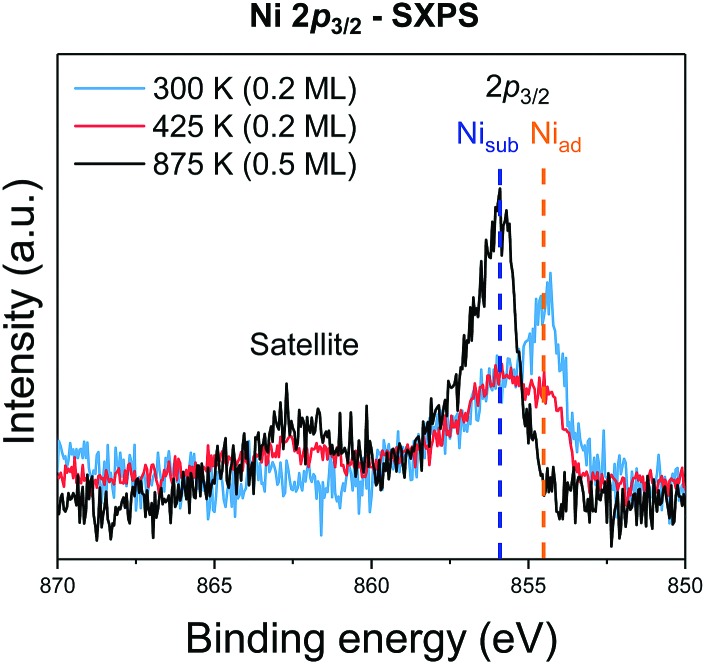
Soft Ni 2p_3/2_ XP spectra (1.1 keV incident photon energy) for as deposited Ni (cyan) and deposited Ni annealed to 425 K and 875 K (red and black respectively). Two distinct species are observed, indicated by the orange and blue dashed lines, and correspond to the Ni_ad_ and Ni_sub_ species, respectively. The underlying Fe 3s has been subtracted from these spectra and they have been normalised to the area of the Ni 2p_3/2_ core level (the original data can be seen in the ESI,† Fig. S3).

Normalised Ni 2p_3/2_ SXP spectra from the first (0.2 ML, 300 K/room temperature), and third (0.5 ML, 875 K) samples are shown in Fig. 2 (the original data can be seen in the ESI,† Fig. S3). Two Ni 2p_3/2_ peaks, with a binding energy difference of 1.4 eV, are present at room temperature and 425 K, but only the higher binding energy peak is present after annealing to 875 K. After annealing, a satellite feature, at even higher binding energy (with a binding energy difference of 7.5 eV to the Ni 2p_3/2_ peak with the lowest binding energy), is also observed. These two chemically distinct Ni species are assigned, as in the work of Bliem *et al.*,^15^ to Ni adatoms (the lower binding energy), Ni_ad_, that are proposed to occupy tetrahedral sites above the surface and incorporated Ni atoms (the higher binding energy), Ni_sub_.^15^ Note that these assignments were based on a comparison of STM images and XP spectra, but are confirmed by the NIXSW data presented here.

Both Ni species are found at a higher binding energy than that of metallic Ni (∼852.8 eV),^19^,^20^ which may indicate a higher oxidation state. In the previous investigation of Cu and Ag adatoms,^18^ DFT calculations predicted a +1 charge state for both adatoms, which was supported by the measured photoelectron binding energy of the Cu 2p state (the Ag binding energies for non-neutral oxidation states are controversial^21^). In contrast, the photoelectron binding energies of the Ni_ad_ and Ni_sub_ species (853.9 eV and 855.2 eV) are more consistent with those of Ni^2+^,^22^ or Ni^3+^,^23^ than the predicted Ni^+^.^15^ While the binding energy of the XP spectra for Ni_ad_ matches that of NiO almost perfectly, assigning the oxidation state on the basis of the photoelectron binding energy implicitly assumes that initial state effects dominate, and ignores potentially important final state effects. As magnetite, in the bulk, is a half-metal, the final-state effects experienced by the Ni adatoms could be very different from those for NiO, which is wide band gap semi-conductor.^24^ Indeed, as detailed in the discussion of the satellite feature in the Ni 2p SXPS, there is good reason to suspect that the electronic structure of Ni_ad_ may differ significantly from that of Ni_sub_, as predicted by Bliem *et al.*^15^

### NIXSW

**Fig. 3:**
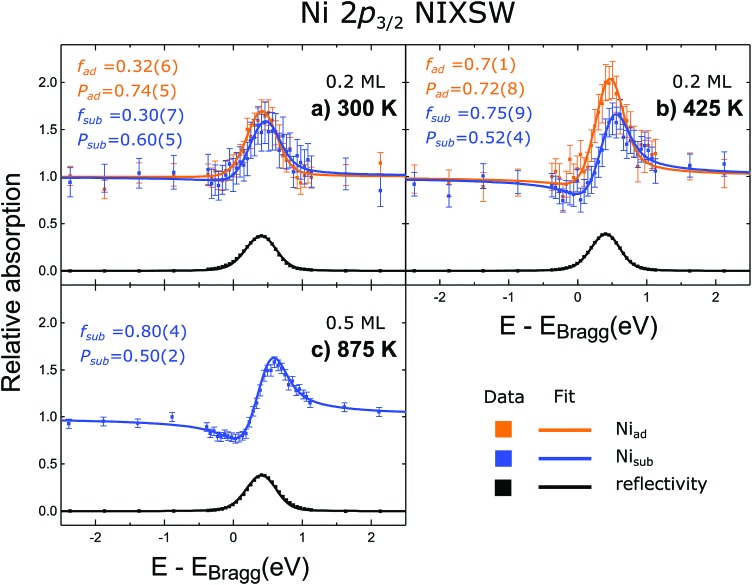
NIXSW photoemission profiles for (a) the as deposited Ni (300 K) and (b–c) the deposited Ni annealed to 425, and 875 K. Both the Ni_ad_ and Ni_sub_ photoemission profiles, and respective P and f values, are given for the as deposited Ni and that annealed to 425 K. Due to the loss of the Ni_ad_ phase at increasing temperatures, and the subsequent weakening of its photoemission peak, no Ni_ad_ photoemission profile could be obtained for the sample annealed to 875 K. These results support the understanding that the Ni_sub_ species is occupying an octahedral site half way between the Fe_tet_ planes.

Representative fitted NIXSW photoemission profiles of Ni_ad_ and Ni_sub_, obtained after annealing to 425 K, are shown in Fig. 3b. The two profiles are very different, indicating that the associated species occupy two significantly different adsorption sites. The resulting coherent fraction and position for the Ni_ad_ species are 0.7 ± 0.1 and 0.72 ± 0.08, respectively. For the Ni_sub_ species, we find values of 0.75 ± 0.09 for the coherent fraction and 0.52 ± 0.04 for the coherent position. These coherent positions result in adsorption heights, with respect to a bulk-like Fe_oct_, of 0.46 ± 0.17 Å for Ni_ad_ and 0.04 ± 0.08 Å for Ni_sub_. Comparable Ni_sub_ spectra, measured after annealing to 875 K (Fig. 3c), show no significant variation in the coherent fraction or position as a function of annealing temperature. Measurements from the as-deposited Ni at room temperature yield much lower values of the coherent fraction for both Ni_ad_ and Ni_sub_ (0.32 ± 0.06 and 0.30 ± 0.07, respectively), indicating significant levels of disorder (most probably due to co-occupation of multiple sites). Prior DFT+U calculations predicted that both a surface octahedral and a sub-surface tetrahedral site are energetically meta-stable (though highly disfavoured),^18^ so at room temperature some Ni atoms may occupy these sites and lack sufficient thermal energy to escape.

**Fig. 4:**
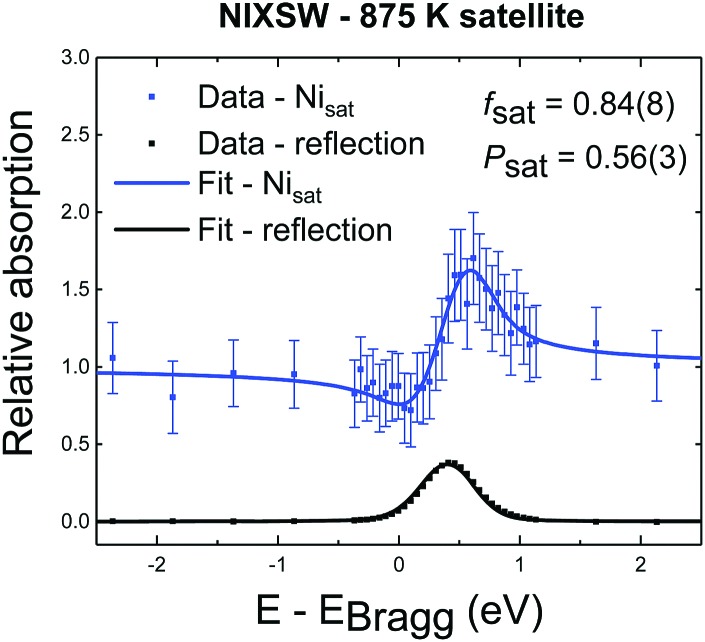
NIXSW photoemission profile for the satellite feature found in the XP spectra of the sample annealed to 875 K. The measured coherent position of 0.56 (±0.03) confirms that this is a loss feature related solely to the Ni_sub_ species.

The satellite feature, which lies at a higher binding energy than the Ni_sub_ elastic peak, has a NIXSW profile (Fig. 4) that is very similar to that of Ni_sub_, suggesting this satellite only arises from Ni atoms in the Ni_sub_ site. It is noteworthy that the coherent fraction of the satellite feature (0.84 ± 0.08) is higher than that of the Ni_sub_ elastic peak, suggesting that not only is the satellite feature only related to Ni_sub_ species, but perhaps even to a specific subset of the Ni_sub_ species.

**Fig. 5:**
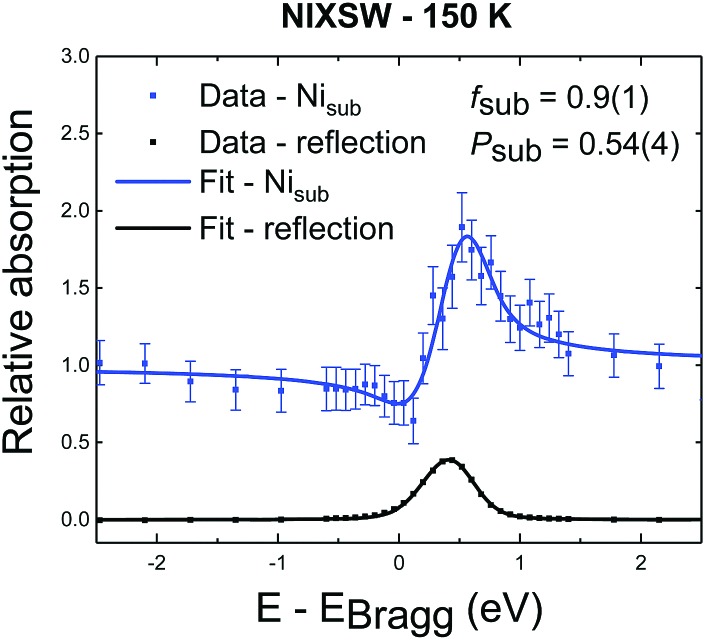
NIXSW photoemission profile for the Ni_sub_ species after depositing 0.3 ML of Ni at 150 K. No appreciable Ni_ad_ phase is found after this low temperature deposition. This is possibly due to the adventitious adsorption of water at such low temperatures and the subsequent loss of the SCV surface reconstruction (see text).

Finally, the additional deposition at ∼150 K was pursued with the aim of creating a higher coverage of Ni_ad_. Contrary to expectations, deposition at this temperature resulted in only Ni_sub_ species being observed in the SXPS with an associated coherent position of 0.54 ± 0.04, that can only be reconciled with complete incorporation of the Ni adatoms, as discussed below. The associated NIXSW profiles are shown in Fig. 5, while SXP spectra are shown in Fig. S4 and S5 (ESI†). Annealing this sample to around room temperature did not significantly alter these results.

### Discussion

The coherent positions of bulk Fe_oct_ layers (*P* = 0.5) and the Ni_sub_ species in all preparations coincide within the experimental uncertainties. To interpret this value as corresponding to adsorption above the surface would require assigning the true height to be one bulk layer spacing larger (*i.e.* the height is (1 + *P*) × *d*_*hkl*_), leading to associated Ni–O bond lengths that would be unphysically long and to a coordination number of the Ni atoms that is too low. Therefore, the NIXSW data clearly demonstrate that Ni_sub_ atoms are indeed incorporated into the magnetite sample (Fig. 1c). This raises two questions: does the incorporated Ni only occupy Fe_oct_ sites, and if so, do these Ni atoms occupy surface or subsurface Fe_oct_ sites?

In the magnetite crystal structure there are two octahedral sites for every one tetrahedral site, and in the bulk crystal the tetrahedral and octahedral sites are antiphase to each other with respect to the (004) standing wave, *i.e.* they differ in coherent position by 0.5. The NIXSW signal from these two sites will therefore interfere destructively, leading to a coherent fraction of 0 if they were equally occupied. With an occupation ratio of octahedral to tetrahedral sites of 2 : 1, as is found in the bulk crystal, the expected coherent fraction would be 0.33. However, the measured coherent fraction for Ni_sub_ is much higher (0.75–0.94), so we can clearly exclude any significant Ni occupation of tetrahedral sites.

Is Ni_sub_ in the surface, or in subsurface octahedral sites? Two consequences arise from the utilisation of the periodic structure of the bulk crystal as a “measuring stick” by the NIXSW technique. The first is that it is not possible to differentiate adsorption heights that differ by an integer value of *d*_*hkl*_, the layer spacing corresponding to the chosen reflection; heights of (*n* + *P*) × *d*_*hkl*_ are equivalent for all values of the integer *n*, and thus a coherent position of 0.5 is indistinguishable from –0.5, +1.5, +2.5 *etc.* The second is that the period and phase of the standing wave are determined by the bulk crystal and are thus insensitive to surface relaxation. For adsorbates, the ambiguity in the value of *n* can usually be easily circumvented, as a difference of ±1 *d*_*hkl*_ in adsorption height will usually result in unrealistic adsorption sites (adsorbate–substrate bond lengths that are too short or too long, as argued above). The problem of surface relaxation can be ignored on most metal surfaces, for which relaxation is typically no more than a few hundredths of an Ångström, but it is not appropriate to neglect this for metal oxides, for which surface relaxation is often much larger.^12^,^25^ In our previous work, studying the adsorption of Cu and Ag adatoms on Fe_3_O_4_(001),^18^ HSE hybrid functional calculations accurately predicted the full adsorption geometry and can therefore provide valuable insight into the probable surface relaxation of this system. The structure predicted by the HSE functional indicated a large inwards relaxation with respect to an ideal bulk termination, of the octahedral iron atoms in the surface layer, by –0.23 Å, –0.22 Å and –0.24 Å for the surface without adatoms, with Cu adatoms and with Ag adatoms respectively (the atomic positions from the DFT calculations for the surface with Ag adatoms and without any adatoms are listed in the ESI† Tables S1 and S2, respectively). A relaxation of this magnitude would shift the coherent position to be expected for the surface octahedral site from 0.5 to 0.38–0.40, significantly lower than that found for Ni_sub_. This would strongly suggest that the Ni_sub_ atoms do not lie in a surface octahedral site, but only occupy sub-surface octahedral sites. The relaxation of the first sub-surface octahedral layer was predicted to be less than our experimental uncertainty (a relaxation of ∼–0.02 Å, compared to an experimental uncertainty of 0.06 Å). Thus, our NIXSW results cannot discriminate between occupation of the first sub-surface octahedral layer and of the bulk. However, the previously published LEED measurements^12^ indicate a loss of the (√2 × √2)*R*45° reconstruction as the concentration of Ni_sub_ species increases. The lifting of the SCV reconstruction does suggest that these Ni_sub_ atoms do occupy some of the sub-surface octahedral vacancies, with other octahedral vacancies possibly occupied by the interstitial Fe_tet_, resulting in the structure shown in Fig. 1c, or by another Ni_sub_ species.

Satellite features in Ni 2p XPS are present in metallic Ni XP spectra, but are assigned to plasmon losses^26^ and are moderately weak. Significantly more pronounced are the satellite features observed in spectra from Ni-ferrites (NiFe_2_O_4_), Ni-oxides and Ni-hydroxides,^26^–^28^ which are assigned to a complex set of multiplets in the final-state electronic structure.^26^ The satellite feature observed here is clearly related only to the Ni_sub_ species, indicating that this species may be very similar electronically to a Ni-ferrite, which would not be surprising as spinel Ni ferrite has the chemical structure of Ni_*x*_Fe_3–*x*_O_4_. What is perhaps more interesting is the converse conclusion that Ni_ad_ is electronically very different from Ni-ferrites, and even other Ni oxides, which also display a rich multiplet structure.^26^ This may suggest that, despite the observed binding energy being significantly higher than that of metallic Ni, the electronic structure of the Ni_ad_ atoms is more similar to metallic Ni than oxidic Ni.

It is not immediately apparent why depositing Ni onto the Fe_3_O_4_ substrate at a significantly lower temperature results in the Ni being driven subsurface. One possible explanation for the near absence of Ni adatoms after deposition onto the surface at low temperature is unintentional adsorption (and partial dissociation) of water. Below 150 K both molecular and dissociative adsorption of water are found to occur on the Fe_3_O_4_(100) surface with molecular water remaining at temperatures up to approximately 240 K.^29^,^30^ The partial pressure of water in the chamber is <3 × 10^–10^ mbar, and the sample was held under these conditions for several minutes before acquisition of SXP spectra. Assuming a unitary sticking coefficient, this would result in an adsorbed density of ∼5 × 10^17^ molecules per m^2^, which is comparable to the adatom density (∼7 × 10^17^ atoms per m^2^). Importantly, the surface dissociation of water, as well as the adsorption of other surface hydroxyl forming species *e.g.* formic acid and atomic hydrogen, is found to lift the (√2 × √2)*R*45° surface reconstruction.^31^,^32^ For metal adatoms that, at high coverage, form nanocluster agglomerates on the magnetite surface (*e.g.* Ag, Pd, and Pt), it is this reconstruction that stabilises the adatom adsorption site by making the di-adatom species energetically unfavourable.^14^ For metal adatoms that incorporate and readily form metal-ferrites, it is possible that the lifting of the reconstruction could result in making the adatom site less favourable and thus promote adsorption in the sub-surface site. How this stabilisation might occur is not immediately clear. It may involve the preference of hydrogen atoms that are dissociated from these molecular species to form hydroxyl species with the oxygen atoms that the Ni adatoms also prefer to bind with,^30^ thus competing the Ni out of the adatom site. Alternately, it may be that the formation of the hydroxyl species makes the octahedral site more energetically favourable or kinetically favourable (*e.g.* lowering the activation barrier between the adatom and the octahedral site). Whatever the mechanism, it could conceivably promote occupation, by Ni atoms, of one (or both) of the sub-surface octahedral vacancy sites. It is worth pointing out that the coherent fraction of the Ni 2p_3/2_ NIXSW, after cold deposition (0.9 ± 0.1), is higher than that after annealing to 875 K (0.8 ± 0.1). This may indicate that, after cold deposition, the Ni atoms only occupy the sub-surface vacancy sites, but after thermal annealing above room temperature, the Ni atoms occupy many different sub-surface/bulk octahedral sites, including the vacancy sites. The potential adsorption of water could be probed by O 1s SXP spectra, however as water was not intentionally dosed on the sample, such spectra were not measured in this study. It was only with hindsight, having identified this surprising result, that the possible role of water was recognised.

## Conclusions

Here we report the adsorption geometry of Ni adsorbed onto Fe_3_O_4_(001), determined by NIXSW. The results of this study confirm previous interpretations of STM images that the deposited Ni atoms occupy both adatom and subsurface sites. Ni adatoms were found to lie significantly lower on the surface than Ag adatoms, but at an almost identical adsorption height to that of Cu adatoms on the same surface. Specifically, the adsorption height of the Ni adatom species was found to be 0.46 ± 0.17 Å above the projected surface Fe_oct_ plane. The subsurface species were found to be co-planar with the Fe_oct_ sites (0.04 ± 0.08 Å above the subsurface Fe_oct_).

The response of Ni adatoms to different annealing temperatures was also studied. After deposition at room temperature, Ni is found to be highly disordered, potentially occupying a range of adsorption sites within the surface region. Annealing the surface results in a more ordered surface layer, where a majority of the Ni atoms occupy either the adatom or the subsurface sites. Upon further annealing, the population of the subsurface species was found to increase at the expense of the Ni adatoms until ultimately all Ni atoms were incorporated into the substrate. As this incorporation leads to a lifting of the SCV reconstruction, it is likely that all of the octahedral vacancies are filled with either Ni atoms and or with Fe_int_ atoms, as indicated in Fig. 1c. It was also found that Ni subsurface atoms dominate after deposition at low temperatures (∼150 K). This may be due to dissociative adsorption of water at these low temperatures leading to loss of the SCV reconstruction and subsequent destabilisation of the adatom phase.

These results support previous extensive STM studies of the same Ni/Fe_3_O_4_(001) surface, in which bright features in the images were observed both between and on the surface Fe_oct_ rows; these were attributed to the existence of adatoms and subsurface atoms, respectively.^15^ Moreover, the temperature at which we find appreciable migration of Ni adatoms into the subsurface cation vacancies to occur (>425 K) is consistent with the calculated temperature at which significant cation defect mobility takes place within Fe_3_O_4_ (∼500 K).^11^

In conclusion, this study provides a direct probe of the cation defect chemistry of the iron oxides and of the importance these defects play in understanding the surface chemistry of Fe_3_O_4_. Ultimately, this study, along with previous work on Cu and Ag adatoms undertaken by the same group, has developed the groundwork for further investigations into the catalytic function of metal/Fe_3_O_4_ surfaces.

## Conflicts of interest

There are no conflicts to declare.

## Supplementary Material

Supplementary information
