# Supplementary material for: Direct measurement of Ni incorporation into Fe3O4(001)
Source: Phys Chem Chem Phys. 2018 Jun 8;20(24):16469–76. doi: 10.1039/c8cp02516a (PMC6011209; doi:10.1039/c8cp02516a)
Supplement: Supplementary file 1 [file CP-020-C8CP02516A-s001.pdf]

Electronic Supplementary Material (ESI) for Physical Chemistry Chemical Physics.
This journal is © the Owner Societies 2018

Direct measurement of Ni incorporation into Fe_3O_4(001)

P. T. P. Ryan[1,2], Z. Jakub[3], J. Balajka[3], J. Hulva[3], M. Meier[3,4], J. T. Küchle[1,5], P. J. Blowey[1,6], P. Thakur Kumar[1], C. Franchini[4], D. J. Payne[2], D. P. Woodruff[6], L. A. Rochford[7], F. Allegretti[5], T. –L. Lee[1], G. S. Parkinson[3] and D. A. Duncan[1]*

[1]Diamond Light Source, Harwell Science and Innovation Campus, Didcot, OX11 0DE, United Kingdom

[2]Department of Materials, Imperial College London, SW7 2AZ, United Kingdom

[3]Institute of Applied Physics, TU Wien, 1040 Vienna, Austria

[4]University of Vienna, Faculty of Physics and Center for Computational Materials Science, 1090 Vienna, Austria

[5]Physics Department E20, Technical University of Munich, 85748 Garching, Germany

[6]Department of Physics, University of Warwick, Coventry, CV4 7AL, United Kingdom

[7]School of Chemistry, University of Birmingham, Birmingham, B15 2TT, United Kingdom

Supplementary Information

Normal incidence X-ray standing wave (NIXSW) XP spectra analysis

One complication in the NIXSW XP spectra data analysis is that the Ni 2*p* and Fe 3*s* photoemission peaks are energetically almost coincident. To remove the influence of the bulk peak a template background was constructed. First XP spectra were measured from a clean SCV reconstructed surface over the binding energy range corresponding to the Ni 2*p* peak, at the same photon energies as the Ni NIXSW measurement. These spectra were summed to produce an integrated spectrum that was then fitted with a Shirley background[1]. The XP Ni 2*p* spectra subsequently recorded for each NIXSW measurement were then fitted utilising this Shirley background-subtracted integrated spectrum (IS) and the Shirley background (SB) itself, in addition to the peaks arising from the presence of the Ni. Both the IS and SB components were scaled by separate linear corrections whose gradient and offset were parameters in the fitting of the XP Ni 2*p* spectra. In part, this fitting procedure is depicted in Figures S1 and S2.

This convoluted analysis, to remove the contribution from the Fe 3*s*, was due to a subtle difference observed in the shape of the background as a function of the photon energy (relative to the Bragg energy), that we attribute to differing absorption profiles of the elastic and inelastic contributions in the background.

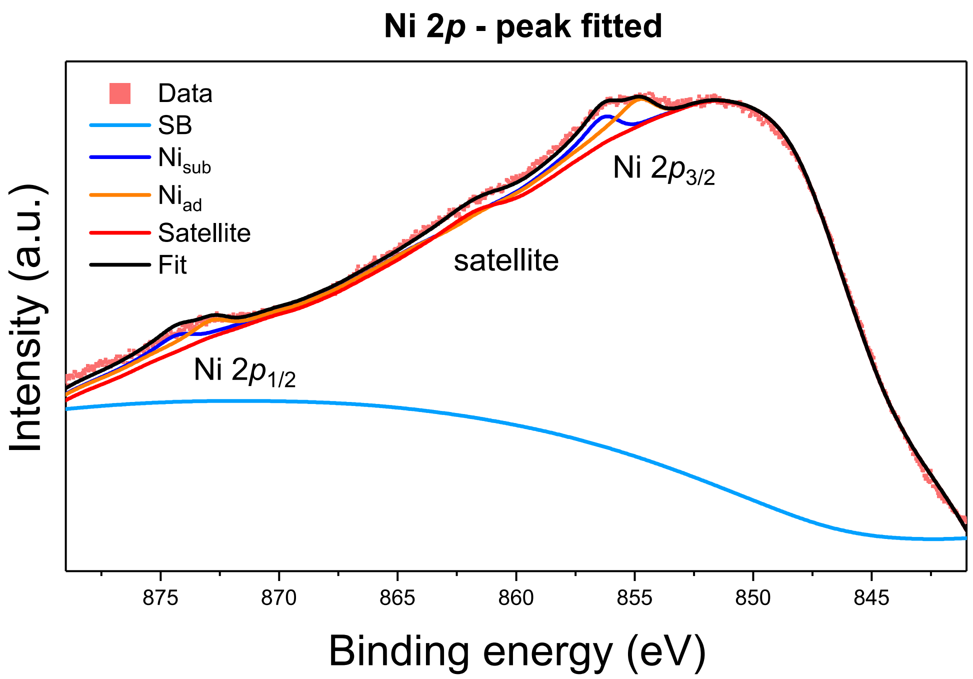

Figure S1 – Depicted is the Ni 2*p* core level with no background subtracted. The large background peak is the bulk Fe 3*s* core level peak. The Shirley background for this Fe 3*s* peak, which was subtracted from the clean background template to give the IS and SB templates as discussed in the text, is depicted in cyan. The Ni_{sub}, Ni_{ad} and satellite fitted peaks are depicted in blue, orange and red respectively. Figure S2 gives an enlarged view of this spectrum in an energy range encompassing only the Ni 2p3/2 doublet.

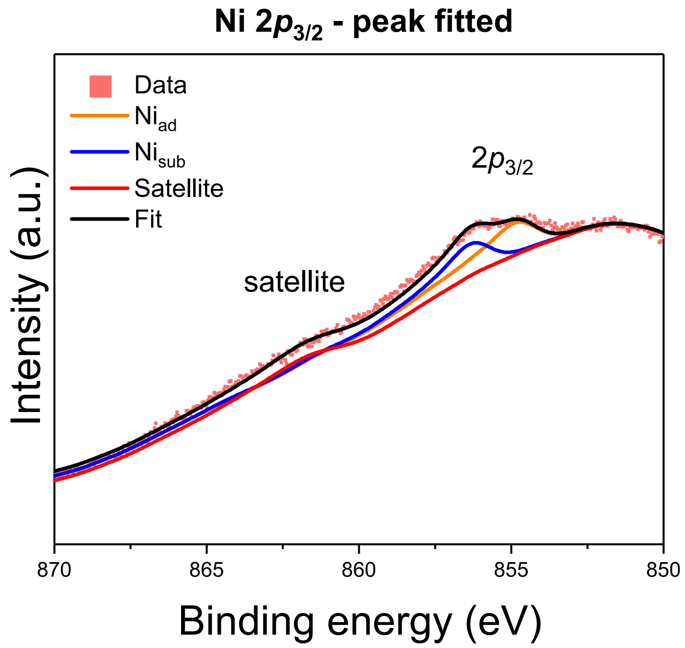

Figure S2 – An enlarged view of Figure S1, only showing the Ni 2$p_{3/2}$ doublet. No background has been subtracted from this spectrum. The Ni_{sub}, Ni_{ad} and satellite fitted peaks are depicted in blue, orange and red respectively.

Soft X-ray photoelectron spectroscopy (SXPS)

Ni $2p_{3/2}$ SXP spectra (hv = 1.1 keV), without background subtraction of the Fe 3*s* SXP spectra, are shown in Figure S1 for Ni deposited onto Fe_3O_4(001) with the substrate held at room temperature and after annealing to 425 and 875 K. Ni $2p_{3/2}$ SXP spectra for Ni deposited onto Fe_3O_4(001) with the substrate held at 150 K, with and without background subtraction, are shown in Figures S2 and S3 (respectively). Figure S2 also shows the Ni $2p_{3/2}$ SXP spectra for Ni deposited onto Fe_3O_4(001) with the substrate held at room temperature, for comparison.

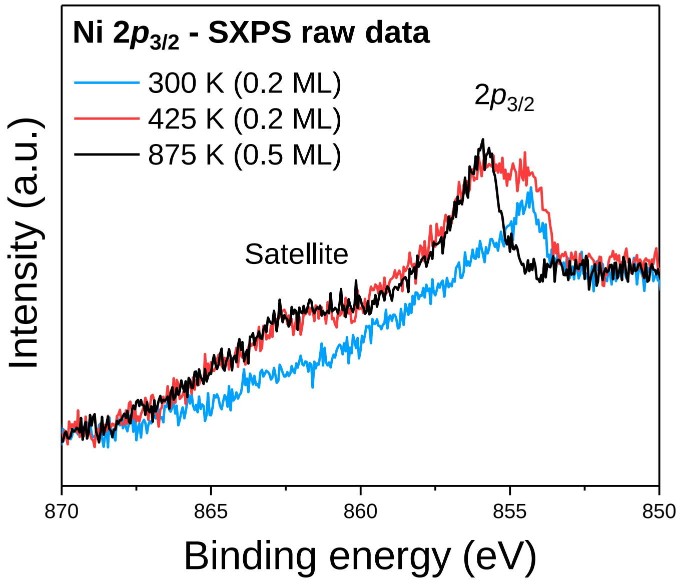

Figure S3 – Raw Ni $2p_{3/2}$ SXP spectra (1.1 keV incident photon energy) for as deposited Ni (blue) and deposited Ni annealed to 425 K and 875 K (red and black respectively). No background has been subtracted from these spectra and they have not been normalised.

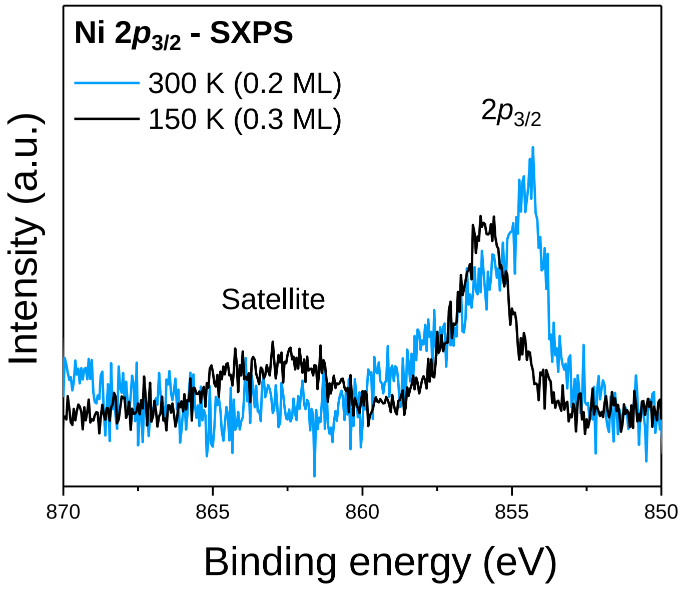

Figure S4 – Ni $2p_{3/2}$ SXP spectra for Ni deposited at 300 K and 150 K. The species present at 150 K is assigned to a subsurface species, as confirmed by the NIXSW results, which exists in conjunction with an adatom species at

300 K. The sole occupation of the subsurface sites by the Ni at 150 K is possibly due to dissociative water adsorption and the subsequent loss of the SCV surface reconstruction.

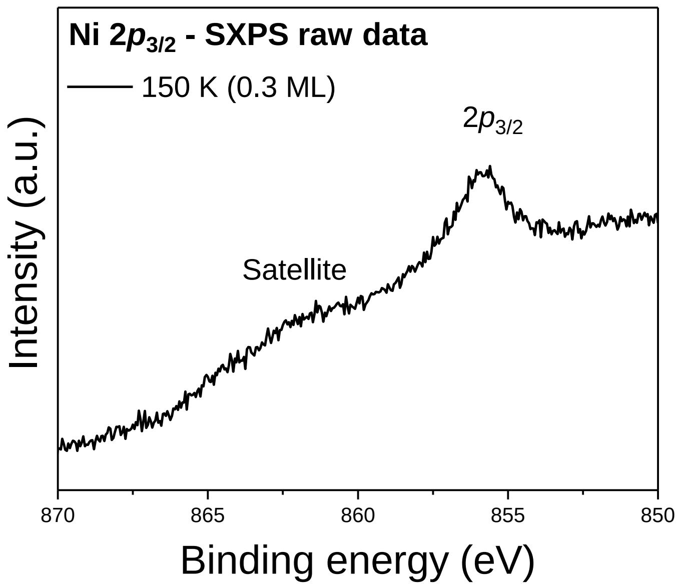

Figure S5 – Raw soft Ni 2$p_{3/2}$ XP spectrum for the Ni metal deposited at 150 K. No background has been subtracted from this spectrum.

Density functional theory (DFT)

Computational details

All the theoretical calculations were performed using the Vienna *ab initio* Simulation Package (VASP)[2,3] using the Projector Augmented Wave (PAW) approach[4,5] with a basis set cut-off energy of 550 eV. The hybrid functional HSE[6] utilised the standard mixing factor 25%, and screening length (0.207^{-1} $Å^{-1}$). The k-mesh of 2 x 2 x 1 was optimised such that it delivers total energy with an accuracy of better than 1 meV, while reducing the k-mesh by a factor of 2 at PBE level leads to a change in the total energy of only 1 meV. In all cases, structures were relaxed until forces were smaller than 0.02 eV/Å.

The surface calculations utilised an asymmetric surface slab (i.e. a slab with a relaxed surface on only a single side), resulting in a significantly cheaper calculation, with 9 fixed layers and 4 relaxed layers with the subsurface cation vacancy reconstruction[7]. Due to large size of the unit-cell (~100 atoms) the adoption of a symmetric setup would be computationally prohibitive for HSE-type calculations. The vacuum gap (separation between adjacent supercells perpendicular to the surface) was set to 14 Å. The resulting relaxed structure for a Ag adatom on Fe_3O_4(001) and a clean Fe_3O_4(001) surface are shown in Tables S1 and S2 (respectively).

Table S1 – Coordinates for the first three octahedrally (Oct) and tetrahedrally (Tet) coordinated layers of the SCV reconstructed surface with an adsorbed Ag adatom, in Å. z = 0 is defined as the 3rd bulk octahedrally coordinated layer. The difference from bulk z positions are shown in the final column (Δz).

layer	atom	x	y	z	Δz
adatom	Ag	1.055	5.261	5.084	-
1st Oct layer	O 1	-0.302	6.604	4.175	-0.00
	O 2	0.142	2.032	4.220	-0.03
	O 3	1.969	0.045	4.191	-0.06
	O 4	2.399	3.904	4.175	-0.00
	O 5	4.181	2.115	4.087	-0.09
	O 6	4.250	6.174	4.191	-0.06
	O 7	6.238	4.348	4.220	-0.03
	O 8	6.321	-0.025	4.087	-0.09
	Fe 1	-0.118	0.145	3.963	-0.25
	Fe 2	2.212	1.939	3.973	-0.24
	Fe 3	4.350	4.088	3.963	-0.25
	Fe 4	6.144	6.417	3.973	-0.24
1st Tet layer	Fe 1	7.453	3.248	3.282	+0.12
	Fe 2	5.258	1.053	3.006	-0.16
(Interstitial)	Fe 3	3.057	7.263	3.225	--
2nd Oct layer	O 1	0.096	-0.114	2.095	+0.03
	O 2	0.137	4.342	2.190	+0.05
	O 3	1.989	2.188	2.096	-0.05
	O 4	1.996	6.202	2.021	-0.05
	O 5	4.091	4.301	2.095	-0.05
	O 6	4.103	-0.102	2.020	-0.05
	O 7	6.393	6.194	2.096	-0.05
	O 8	6.419	2.214	2.041	-0.03
	Fe 1	-0.083	6.342	2.085	-0.02
	Fe 2	2.136	4.122	2.085	-0.02
2nd Tet layer	Fe 1	1.026	1.049	1.033	-0.02
	Fe 2	5.254	5.231	1.033	-0.02
3rd Oct layer	O 1	-0.038	2.141	-0.038	--
	O 2	0.038	6.270	0.038	--
	O 3	2.065	4.243	0.038	--
	O 4	2.141	-0.038	-0.038	--
	O 5	4.167	6.346	-0.038	--
	O 6	4.243	2.065	0.038	--
	O 7	6.270	0.038	0.038	--
	O 8	6.346	4.167	-0.038	--
	Fe 1	0.000	4.205	0.000	--
	Fe 2	2.103	6.308	0.000	--
	Fe 3	4.205	0.000	0.000	--
	Fe 4	6.308	2.103	0.000	--
3rd Tet layer	Fe 1	3.154	3.154	-1.051	--
	Fe 2	7.359	7.359	-1.051	--

Table S2 – Coordinates for the first three octahedrally (Oct) and tetrahedrally (Tet) coordinated layers of the SCV reconstructed surface, in Å. z = 0 is defined as the 3rd bulk octahedrally coordinated layer. The difference from bulk z positions are shown in the final column (Δz).

layer	atom	x	y	z	Δz
1st Oct layer	O 1	-0.248	6.561	4.068	-0.11
	O 2	0.133	2.029	4.166	-0.09
	O 3	1.978	0.079	4.168	-0.08
	O 4	2.355	3.958	4.068	-0.11
	O 5	4.177	2.137	4.091	-0.08
	O 6	4.285	6.183	4.168	-0.08
	O 7	6.234	4.338	4.166	-0.09
	O 8	6.343	-0.029	4.091	-0.08
	Fe 1	-0.086	0.116	3.975	-0.24
	Fe 2	2.196	1.992	3.974	-0.24
	Fe 3	4.322	4.119	3.975	-0.24
	Fe 4	6.197	6.401	3.974	-0.24
1st Tet layer	Fe 1	5.258	1.053	3.032	-0.13
	Fe 2	7.457	3.251	3.254	+0.09
(Interstitial)	Fe 3	3.060	7.265	3.255	-
2nd Oct layer	O 1	0.093	4.298	1.996	-0.07
	O 2	0.109	-0.102	2.076	-0.07
	O 3	2.003	2.208	2.074	-0.07
	O 4	2.012	6.217	1.997	-0.07
	O 5	4.103	4.314	2.076	-0.07
	O 6	4.107	-0.098	2.014	-0.05
	O 7	6.408	2.202	2.016	-0.05
	O 8	6.413	6.209	2.074	-0.07
	Fe 1	-0.052	6.364	2.167	+0.06
	Fe 2	2.159	4.154	2.167	+0.06
2nd Tet layer	Fe 1	1.051	1.056	1.056	0.00
	Fe 2	5.261	5.257	1.056	0.00
3rd Oct layer	O 1	-0.038	2.141	-0.038	--
	O 2	0.038	6.270	0.038	--
	O 3	2.065	4.243	0.038	--
	O 4	2.141	-0.038	-0.038	--
	O 5	4.167	6.346	-0.038	--
	O 6	4.243	2.065	0.038	--
	O 7	6.270	0.038	0.038	--
	O 8	6.346	4.167	-0.038	--
	Fe 1	0.000	4.205	0.000	--
	Fe 2	2.103	6.308	0.000	--
	Fe 3	4.205	0.000	0.000	--
	Fe 4	6.308	2.103	0.000	--
3rd Tet layer	Fe 1	3.154	3.154	-1.051	--
	Fe 2	7.359	7.359	-1.051	--

1 D. A. Shirley, *Phys. Rev. B*, 1972, **5**, 4709–4714.

2 G. Kresse and J. Hafner, *Phys. Rev. B*, 1993, **48**, 13115–13118.

3 G. Kresse and J. Furthmüller, *Comput. Mater. Sci.*, 1996, **6**, 15–50.

4 P. E. Blöchl, *Phys. Rev. B*, 1994, **50**, 17953–17979.

5 D. Joubert, *Phys. Rev. B - Condens. Matter Mater. Phys.*, 1999, **59**, 1758–1775.

6 J. Heyd, G. E. Scuseria and M. Ernzerhof, *J. Chem. Phys.*, 2003, **118**, 8207–8215.

7 R. Bliem, E. McDermott, P. Ferstl, M. Setvin, O. Gamba, J. Pavelec, M. A. Schneider, M. Schmid, U. Diebold, P. Blaha, L. Hammer and G. S. Parkinson, *Science*, 2014, **346**, 1215–1218.
